# Editorial on Special Issue “Artificial Intelligence in Image-Based Screening, Diagnostics, and Clinical Care”

**DOI:** 10.3390/diagnostics14171984

**Published:** 2024-09-07

**Authors:** Sivaramakrishnan Rajaraman, Zhiyun Xue, Sameer Antani

**Affiliations:** Computational Health Research Branch, National Library of Medicine, National Institutes of Health, Bethesda, MD 20894, USA; sivaramakrishnan.rajaraman@nih.gov (S.R.); zhiyun.xue@nih.gov (Z.X.)

## 1. Introduction

In an era of rapid advancements in artificial intelligence (AI) technologies, particularly in medical imaging and natural language processing, strategic efforts to leverage AI’s capabilities in analyzing complex medical data and integrating it into clinical workflows have emerged as a key driver of innovation in healthcare. As the global healthcare landscape shifts towards precision medicine, the incorporation of AI into image-based screening, diagnostics, and clinical care is becoming increasingly crucial, offering significant opportunities to enhance patient outcomes and improve healthcare delivery. Advancements in AI, particularly in deep learning (DL) [1], a subset of machine learning (ML), have significantly advanced medical imaging, and accurate analyses. These developments represent a paradigm shift, where AI not only automates processes but also enhances precision in diagnostics, allowing for personalized interventions tailored to individual patient needs. However, integrating AI into clinical workflow presents several challenges that must be carefully addressed. The effectiveness and generalizability of AI-driven solutions can be adversely affected by the data quality, imbalance, and limited availability of well-annotated training data [2]. The imbalance in datasets [3] refers to pathological cases in various grades of severity that are significantly smaller compared to healthy controls. These factors complicate the development of robust, reliable, bias-free, and generalizable models and limit their use in real-world clinical environments.

As Guest Editors of the Special Issue “Artificial Intelligence in Image-Based Screening, Diagnostics, and Clinical Care”, we present a collection of research findings addressing some of these challenges, showcasing cutting-edge advancements in AI applications in healthcare. This Special Issue not only highlights technological progress but also explores the practical implications of AI in clinical practice, offering insights that are critical for the ongoing evolution of the field. We believe that the contributions within this Special Issue will serve as a catalyst for future research and encourage the broader medical and scientific communities to fully explore AI’s potential in transforming patient care.

## 2. Highlights of the Special Issue

### 2.1. Overview of Published Research

This Special Issue “Artificial Intelligence in Image-Based Screening, Diagnostics, and Clinical Care” offers a comprehensive collection of 12 research studies that explore various AI methodologies, each contributing to the advancement of precision medicine and the improvement of clinical care.

### 2.2. AI in Cardiac Diagnostics

Cardiac diagnostics are crucial areas where AI has demonstrated significant impact, particularly in enhancing the accuracy and efficiency of clinical workflows. The study “Novel Domain Knowledge-Encoding Algorithm Enables Label-Efficient Deep Learning for Cardiac CT Segmentation to Guide Atrial Fibrillation Treatment in a Pilot Dataset” [4] addresses a critical challenge in AI-driven medical imaging: the need for large, labeled datasets. The authors propose a novel methodology that encodes domain-specific cardiac geometry knowledge to automate the labeling process, thereby reducing the dependency on extensive training data. This innovative approach achieved high segmentation accuracy in cardiac CT images with minimal training data, highlighting the potential for AI to improve personalized treatment strategies, such as cardiac ablation for atrial fibrillation, where precise segmentation of cardiac structures is essential.

Complementing this, the study “AI-Driven Real-Time Classification of ECG Signals for Cardiac Monitoring Using i-AlexNet Architecture” [5] focuses on the application of AI in real-time cardiac monitoring. The authors developed a new model called the *i-AlexNet* model, a modified version of the classic AlexNet [6] architecture, which excels in classifying ECG signals with remarkable accuracy. With a classification accuracy of 98.8%, this study not only underscores the robustness of AI in cardiac monitoring but also highlights its potential to revolutionize real-time, data-driven healthcare solutions.

AI’s ability to accurately classify normal and anomalous ECG signals in real time can greatly assist clinicians in making timely and accurate decisions, ultimately improving patient outcomes. The review article “Revolutionizing Cardiology through Artificial Intelligence—Big Data from Proactive Prevention to Precise Diagnostics and Cutting-Edge Treatment—A Comprehensive Review of the Past 5 Years” [7] discusses findings from multiple studies, exploring how AI is being integrated into various branches of cardiology, including imaging, electrophysiology, and interventional procedures. This comprehensive review not only highlights the rapid advancements in AI technologies and their potential to revolutionize cardiovascular care but also addresses the ethical and legal challenges associated with their implementation.

The study “Diagnostic AI and Cardiac Diseases” [8] further explores the diagnostic applications of AI for cardiac conditions. The authors review AI-driven tools for detecting various cardiac diseases, emphasizing the importance of AI as a support system for clinical decision making. By categorizing the reviewed studies according to specific cardiac conditions, the article provides a structured overview of how AI is enhancing diagnostic accuracy and improving patient outcomes.

The study “Developing a Deep-Learning-Based Coronary Artery Disease Detection Technique Using Computer Tomography Images” [9] presents an advanced DL model for coronary artery disease (CAD) detection. By utilizing YOLOV7 [10] for feature extraction and optimizing the hyperparameters of the UNet++ model, the authors created a CAD detection system that surpasses current methods, highlighting AI’s potential for fine-tuning across different medical imaging applications, including oncology.

### 2.3. AI in Chest X-ray (CXR) Analysis

Chest X-ray (CXR) image analysis represents another critical area where AI is making significant strides, particularly in the context of detecting infectious diseases and chronic conditions. A significant portion of the Special Issue is dedicated to the application of AI in CXR analysis. The study “Deep Neural Network Augments Performance of Junior Residents in Diagnosing COVID-19 Pneumonia on Chest Radiographs” [11] demonstrates how AI can enhance clinical decision making, especially for less experienced practitioners. The authors developed a DL network capable of distinguishing COVID-19 pneumonia from other types of pneumonia, significantly improving the diagnostic accuracy of junior residents. This research underscores AI’s potential as an educational tool, bridging the experience gap and improving the overall quality of care in high-pressure situations.

In addressing domain-specific challenges, the study “Cross Dataset Analysis of Domain Shift in CXR Lung Region Detection” [12] investigates the impact of domain shift, a phenomenon where differences in data sources can affect AI model performance. By analyzing five CXR datasets from different sources, the authors provide insights into how to mitigate domain shifts and enhance the robustness of AI models in diverse clinical environments. This study contributes to the broader goal of developing AI models that can be generalized across various settings, a key concern for the real-world deployment of AI in healthcare.

The study titled “Assessing the Impact of Image Resolution on Deep Learning for TB Lesion Segmentation on Frontal Chest X-rays” [13] investigates the influence of image resolution on the effectiveness of DL models in segmenting lesions consistent with tuberculosis (TB) in CXRs. An Inception-V3 encoder-based UNet [14] model was systematically evaluated in this segmentation task using image-mask pairs across various spatial resolutions, identifying the optimal resolution for accurate TB lesion segmentation. This research is particularly relevant for developing AI models that are both computationally efficient and diagnostically accurate, especially in resource-limited settings where high-resolution imaging may not be feasible.

Expanding on the application of AI in CXR analysis, the study “Analysis of Chest X-ray for COVID-19 Diagnosis as a Use Case for an HPC-Enabled Data Analysis and Machine Learning Platform for Medical Diagnosis Support” [15] demonstrates how high-performance computing (HPC) can accelerate the development of AI tools for real-time clinical applications. By leveraging an HPC platform, the authors re-trained the COVID-Net [16] model, optimizing its performance through large-scale hyperparameter tuning. This study highlights the importance of computational resources in AI development, particularly in responding to global health crises like the COVID-19 pandemic.

The article titled “Performance and Agreement When Annotating Chest X-ray Text Reports—A Preliminary Step in the Development of a Deep Learning-Based Prioritization and Detection System” [17] addresses the crucial issue of consistency in data annotation. The study investigates how different annotators, ranging from radiologists to medical students, interpret and label CXR radiological reports. The findings reveal notable variability in annotation quality, which directly impacts the development of reliable AI-based decision support systems. By emphasizing the importance of consistent and accurate annotations, this research contributes to the ongoing discourse on the role of human expertise in training AI models, particularly in fields where sub-specialization is common. Additionally, the article “Inter- and Intra-Observer Agreement When Using a Diagnostic Labeling Scheme for Annotating Findings on Chest X-rays” [18] explores the variability in annotation consistency among radiologists, a key factor in the successful deployment of AI systems. The study evaluates how experience levels affect annotation consistency, providing valuable insights into the challenges of standardizing data for AI training. The findings suggest that descriptive labels, as opposed to interpretive ones, may increase agreement among radiologists, thereby improving the reliability of AI-based decision support systems.

### 2.4. AI in Oncology: Lung Cancer Detection

Oncology, particularly lung cancer detection, is another focal point of this Special Issue, where AI’s potential to enhance early detection and treatment outcomes is thoroughly explored. The study “Diagnostic Accuracy of Machine Learning AI Architectures in Detection and Classification of Lung Cancer: A Systematic Review” [19] provides a comprehensive assessment of various ML/DL architectures used in lung cancer detection. The review analyzes multiple studies, highlighting the sensitivity, specificity, and overall accuracy of these models in distinguishing between malignant and benign lung lesions. This systematic review underscores the potential of AI to significantly improve early detection and classification of lung cancer, a critical factor in improving patient prognosis. The study also emphasizes the need for further research to optimize and validate AI algorithms, ensuring their clinical relevance and applicability in routine practice.

### 2.5. Innovative Approaches

The studies featured in this Special Issue collectively demonstrate innovative approaches to addressing the challenges associated with limited and imperfect medical data. For instance, the methods proposed in [4] demonstrate a notable advancement in reducing the dependency on large datasets by incorporating domain knowledge directly into the AI training process. This method not only enhances model efficiency but also opens new avenues for AI applications in areas where data scarcity is a limiting factor. The integration of HPC, as seen in [15], represents a forward-looking approach to accelerating AI development, making it possible to deploy highly optimized models in real-time clinical settings. This innovation is particularly relevant in the context of global health emergencies, where rapid and accurate diagnostics are paramount. Moreover, studies [17,18] on exploring the annotation consistency in CXR labeling highlight the human factors that must be considered in AI development. The review articles on AI in cardiology [7,8] and lung cancer detection [19] further illustrate the broad applicability of AI across different medical fields. By discussing recent advancements and identifying areas for future research, these reviews offer a roadmap for the continued integration of AI into clinical practice.

## 3. Conclusions

### 3.1. Summary of Key Points

In this Special Issue on “Artificial Intelligence in Image-Based Screening, Diagnostics, and Clinical Care”, we have examined the diverse and transformative role of AI across a wide range of medical applications. The 12 research studies featured in this issue have highlighted the potential of AI to enhance precision medicine, improve diagnostic accuracy, and streamline clinical workflows.

### 3.2. Call to Action

As we look to the future, the medical and scientific communities must continue to explore and expand the possibilities of AI in healthcare. The advancements discussed in this Special Issue are just the beginning. There remains a wealth of untapped potential in AI technologies, particularly in the areas of multi-modal data integration, explainable AI, and bias mitigation. Future research must not only focus on pushing the boundaries of what AI can achieve but also on ensuring that these technologies are developed and deployed in ways that are ethical, equitable, and centered around patient care. Collaboration across disciplines will be key to achieving these goals. AI researchers, clinicians, data scientists, ethicists, and policymakers must work together to create AI systems that are not only optimal but also aligned with the needs and values of the healthcare community. By fostering a collaborative and interdisciplinary approach, we can ensure that AI continues to evolve as a tool that enhances, rather than replaces, the expertise and judgment of human clinicians.

The studies included here represent a significant contribution to the field of AI in medical data analysis, but there is much more to be done. By engaging with this Special Issue and contributing to this field, you become part of a growing community dedicated to exploring the frontiers of AI in healthcare. Together, we can push the boundaries of what is possible, improving patient outcomes and making high-quality healthcare more accessible to people around the world.
